# Abyssal seafloor as a key driver of ocean trace-metal biogeochemical cycles

**DOI:** 10.1038/s41586-025-09038-3

**Published:** 2025-06-11

**Authors:** Jianghui Du, Brian A. Haley, James McManus, Patrick Blaser, Jörg Rickli, Derek Vance

**Affiliations:** 1 Institute of Geochemistry and Petrology, Department of Earth and Planetary Sciences, ETH Zürich, Zürich, Switzerland; 2 Key Laboratory of Orogenic Belts and Crustal Evolution, MOE; School of Earth and Space Sciences, Peking University, Beijing, China; 3 College of Earth, Ocean and Atmospheric Sciences, Oregon State University, Corvallis, OR USA; 4 Bigelow Laboratory for Ocean Sciences, East Boothbay, ME USA; 5 Institute of Earth Sciences, University of Lausanne, Lausanne, Switzerland; 6 GEOMAR Helmholtz-Zentrum für Ozeanforschung Kiel, Kiel, Germany

**Keywords:** Marine chemistry, Element cycles

## Abstract

Trace elements and isotopes (TEIs) are important to marine life and are essential tools for studying ocean processes^1^. Two different frameworks have arisen regarding marine TEI cycling: reversible scavenging favours water-column control on TEI distributions^2–5^, and seafloor boundary exchange emphasizes sedimentary imprints on water-column biogeochemistry^6,7^. These two views lead to disparate interpretations of TEI behaviours^8–10^. Here we use rare earth elements and neodymium isotopes as exemplar tracers of particle scavenging^11^ and boundary exchange^6,7,12^. We integrate these data with models of particle cycling and sediment diagenesis to propose a general framework for marine TEI cycling. We show that, for elements with greater affinity for manganese oxide than biogenic particles, scavenging is a net sink throughout the water column, contrary to a common assumption for reversible scavenging^3,13^. In this case, a benthic flux supports increasing elemental concentrations with water depth. This sedimentary source consists of two components: one recycled from elements scavenged by water-column particles, and another newly introduced to the water column through marine silicate weathering inside sediment^8,14,15^. Abyssal oxic diagenesis drives this benthic source, and exerts a strong influence on water-column biogeochemistry through seafloor geometry and bottom-intensified turbulent mixing^16,17^. Our findings affirm the role of authigenic minerals, often overshadowed by biogenic particles, in water-column cycling^18^, and suggest that the abyssal seafloor, often regarded as inactive, is a focus of biogeochemical transformation^19,20^.

## Abstract (web summary)

Rare earth element and neodymium isotope data combined with models of particle cycling and sediment diagenesis suggest that the abyssal seafloor is a key driver of trace-metal biogeochemical cycles.

## Main

Many trace elements are essential nutrients for marine life and have a key role in the carbon cycle^1^. They are also indispensable tools for investigating the evolution of the ocean and climate systems in Earth history^1^. Studying their marine cycles is vital for safeguarding marine ecosystems and understanding past and current climate change. Historically, interpretations of trace-element cycles have followed a top-down ‘nutrient-type’ model^21^. Reversible scavenging, a leading mechanism, proposes that the decreasing concentration of scavenging particles with depth releases surface-sourced elements^3–5^. This mechanism was first applied to thorium (Th) isotopes^2^; however, they have radioactive decay sources in the water column to support their concentration increase with depth. For other elements, ocean biogeochemical models (OBMs) generally assume that remineralization of biogenic particles provides the internal source^3–5^. Such models often ignore manganese (Mn) and iron (Fe) oxides, despite the fact that they are established scavengers^11,22^, as evidenced in the latest GEOTRACES studies^23–26^.

Recent discoveries of widespread sedimentary imprints on the distributions of many trace elements and isotopes (TEIs), such as Fe (refs. ^27,28^), neodymium (Nd)^6,8,9^, beryllium (Be)^10^, lead (Pb)^29^, nickel (Ni)^30^, copper (Cu)^31^, chromium (Cr)^32^ and their isotopes, are discordant with the top-down view. An alternative boundary-exchange view argues that particle-dissolved exchange at ocean boundaries drives trace-element cycles^6,7,12^. A bottom-up diagenetic benthic flux is a favoured candidate to explain boundary exchange, supported by observational^10,19,20,28,32,33^ and modelling evidence^13,15,31,34^. But it remains unclear what processes facilitate sedimentary influences on the water column and how the benthic flux is linked to particle scavenging.

Critically, the top-down and bottom-up models can imply completely different behaviours of TEIs as tracers of ocean processes. For example, seawater radiogenic Nd isotope composition (*ε*_Nd_) has been used as a conservative water-mass mixing tracer assuming that external sources of Nd exist only at the surface^35^; however, recent arguments for a strong benthic control propose *ε*_Nd_ as a non-conservative tracer of abyssal circulation rate^9,33,36^, or in extreme cases, a proxy of local sediment provenance^8,9^. Understanding the relative importance of top-down versus bottom-up processes is thus essential.

So far, studies of particle scavenging and boundary exchange are heavily biased towards the upper ocean, where biogenic particles are most abundant, and continental margins, where reducing sediment environments often dominate. However, emerging evidence based on seawater Fe (ref. ^37^) and Nd (ref. ^9^) isotopes and porewater geochemistry^20,33^ suggests that the oxic abyssal seafloor, long overlooked, can host important biogeochemical transformations. Moreover, recent ocean-mixing studies have revealed that the abyssal ocean generates surprisingly strong turbulence through interaction of internal tides with seafloor topography, creating bottom-intensified diapycnal mixing that sustains the overturning circulation^16,17^. Considering the areal extent of this benthic mixing, any geochemical exchange at the abyssal seafloor should be quantitatively important.

Here we report water-column, sediment and porewater data from the abyssal central Pacific. We use these data to constrain a model that constitutes an integrated framework for describing marine trace-element cycling from the water column to abyssal sediment that stresses the importance of benthic impacts. We use rare earth elements (REE; including Nd) and Nd isotopes to constrain the model, exploiting their proven utility in studying particle scavenging, ocean mixing and boundary exchange^6,7,11,35^. Neodymium has the advantage that its residence time is relatively short (about 400–500 yr)^9^, such that circulation does not mask the impacts of particle scavenging and benthic flux; but long enough to be a tracer of basinal to global, rather than local, processes. We use these tracers to develop a broader concept: the interplay between particle scavenging and benthic processing is relevant to many trace elements. We focus on the abyssal Pacific, a quarter of Earth’s surface area, whose weak overturning allows the impact of biogeochemical cycling to manifest most clearly.

## Particulate carriers

Marine particles are categorized into biogenic, lithogenic and authigenic types^22,25^. For example, biogenic particles include soft tissues and mineral shells (for example, carbonate and opal), each with different processes controlling the nature and length scales of remineralization^3,13^. Authigenic phases, precipitating from seawater or pore water, are best exemplified by Mn and Fe (oxyhydr)oxides, as macroscopic structures (for example, nodules) or microscopic coatings on other particles^22^. Marine particles in nature are a mix of all three types, and thus isolating them into their respective fractions is needed to better understand scavenging dynamics.

**Fig. 1:**
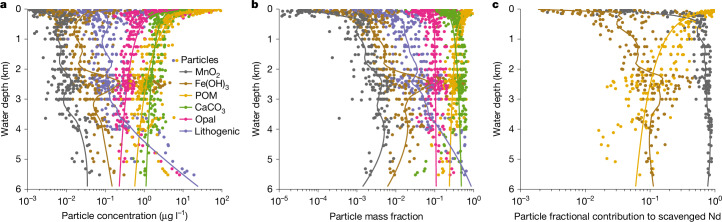
Particle scavenging in the Pacific. **a**,**b**, Particle concentration (**a**) and mass fraction (**b**) from the Pacific GEOTRACES data^25,38^. **c**, Estimated particulate contribution to scavenged Nd using data-constrained *K*_d_; carbonate, opal and lithogenic particles are not significant scavengers and are not shown (see ‘Particle scavenging’ in Methods). Despite being the least abundant scavenging phase, authigenic Mn oxide makes the predominant contribution to Nd scavenging. Lines in the plots are either power-law (biogenic particles) or locally estimated scatterplot smoothing (LOESS) (other particle types) trends with depth.

We quantitatively attributed the association of scavenged REE using partition coefficients^23–26^ (*K*_d_) estimated with the global GEOTRACES data^38^ (see ‘Particle scavenging’ in Methods). The result shows that Nd has the greatest affinity for Mn oxide (log_10_*K*_d_, 8.76 median value, 8.72–8.79 interquartile range), followed by Fe oxide (7.26, 7.21–7.37), both being much greater than particulate organic matter (POM; 5.93, 5.86–6.01), whereas carbonate, opal and lithogenic particles are probably insignificant especially in the deep ocean, consistent with previous regional studies^23,26^. This affinity pattern is similar to that of Th, protactinium (Pa), polonium (Po) and Pb (refs. ^24,39^), demonstrating the potency of Mn oxide in scavenging a wide range of TEIs. Beneath the surface, Mn oxide makes up less than 1% of the particle mass but accounts for approximately 50–90% of scavenged Nd in the Pacific (Fig. 1).

**Fig. 2:**
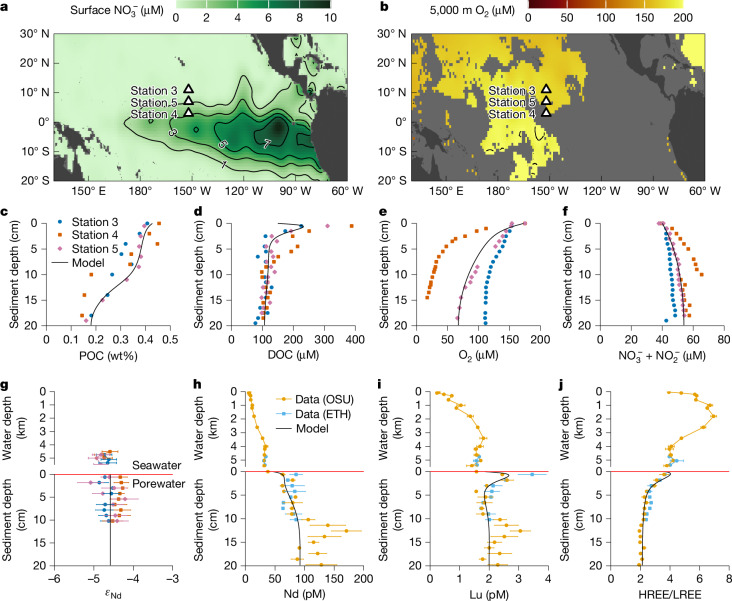
Pacific sites and biogeochemistry. **a**, Surface-ocean nitrate concentrations^56^. **b**, Oxygen concentration at 5,000 m water depth^56^. **c**–**f**, Concentrations of sediment particulate organic carbon (POC; **c**), porewater dissolved organic carbon (DOC; **d**), oxygen (O_2_; **e**) and nitrate + nitrite (NO_3_^−^ + NO_2_^−^; **f**). **g**–**j**, Seawater and porewater profiles of *ε*_Nd_ (with 2*σ* error bars; **g**), Nd (**h**) and Lu (**i**) concentrations, and HREE/LREE ratios (that is, [Tm + Yb + Lu]/[La + Pr + Nd] using shale-normalized concentrations; **j**). In **c**–**g**, data are measured in the same lab and we plot the three sites separately (see ‘Pacific cruise’ in Methods). In **h**–**j**, data are measured at both Oregon State University (OSU) and ETH Zurich (ETH) and we show the mean values of the three sites and the standard errors of the mean values. The black lines in **c**–**j** are modelled values. See ‘Diagenetic model’ in Methods and Extended Data Figs. 2 and 3 for detailed model description and results.

**Extended Data Fig. 1:**
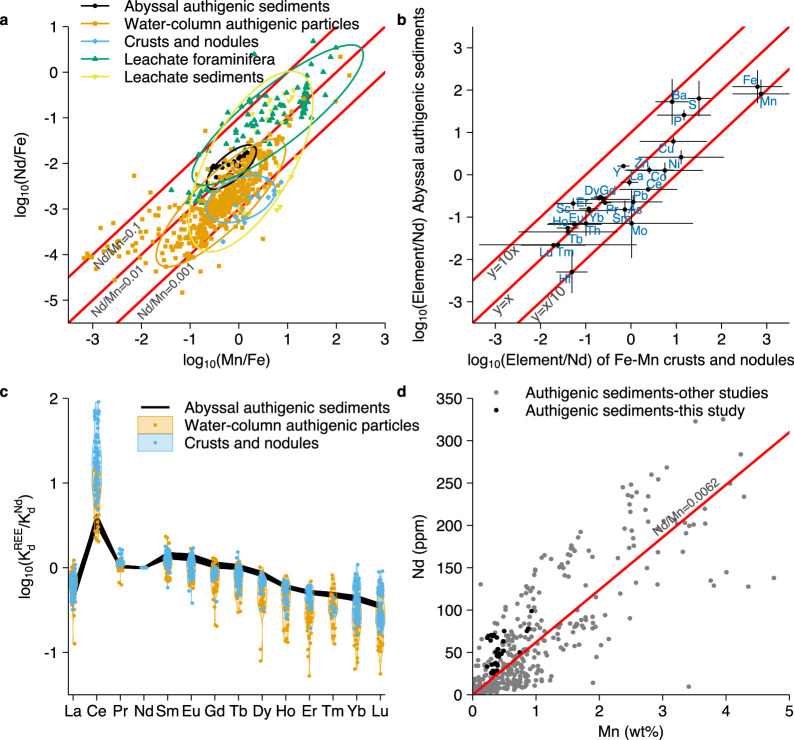
Elemental composition of abyssal authigenic sediments and water-column authigenic particles compared with natural oxide rich phases. **a**, The Nd/Fe ratio vs. Mn/Fe ratio in authigenic sediments, authigenic particles, Fe-Mn crusts and nodules, extractions of Fe-Mn oxyhydroxides by leaching marine sediments and foraminifera. The red lines indicate constant Nd/Mn ratios that range from 10^−3^ to 10^−1^. **b**, Element/Nd ratios in authigenic sediments compared with those in crusts and nodules. The red lines show the 1-order of magnitude envelope around the 1:1 line. **c**, Partition coefficients of REE (relative to that of Nd) in authigenic sediments compared with authigenic particles, and crusts and nodules. Particle, crust and nodule data, in coloured dots, are summarized using the violin plot to show the statistical distributions. **d**, Correlation between Nd and Mn concentration in abyssal sediments from this study and other studies in the Pacific. Data sources to the plots are summarized in Methods: REE carriers.

Our abyssal (below 5,000 m water depth) sediment data agree with this water-column result. From the biogenic-rich Station 4 at the border of the productive equatorial upwelling zone, to the red-clay-site Station 3 underlying the oligotrophic gyre (Fig. 2a and ‘Pacific cruise’ in Methods), sediment REE are dominantly found in authigenic fractions (see ‘Abyssal Pacific sediment’ in Methods), including 76 ± 9% Nd (1*σ*). The consistency of authigenic enrichment across our sites, despite an approximately fivefold change of sinking biogenic particle flux^40^, shows that biogenic particles are negligible carriers of REE. Authigenic sediments also account for 89 ± 6% of Mn but for only 26 ± 16% of Fe. The authigenic Mn/Fe ratio (0.3–1.3, 95% range) is much higher than for the upper continental crust^41^ (0.02), suggesting the prevalence of Mn oxide. The geochemical compositions of water-column authigenic particles and abyssal authigenic sediments are similar, both resembling those of oxide-rich phases (Extended Data Fig. 1 and ‘REE carriers’ in Methods). Such similarity points to the highly consistent geochemical nature of oxides across a wide range of environmental conditions.

We further found an agreement between the particle rain and sediment burial rates of REE, establishing the consistency of water-column-to-sediment mass transfer. Combining published particle concentration data with sediment-trap-based mass flux data in the study region (see ‘Water-column and sediment mass fluxes’ in Methods), we estimate that the rain rates of authigenic and total particulate Nd are 55 ± 24 (1*σ*) pmol cm^−2^ yr^−1^ and 68 ± 27 pmol cm^−2^ yr^−1^, respectively, agreeing with the estimated burial rates of 53 ± 17 pmol cm^−2^ yr^−1^ and 62 ± 20 pmol cm^−2^ yr^−1^, respectively.

Integrating these independent lines of evidence, we conclude that Mn oxide is the dominant host of water-column and surface-sediment REE. Essentially, abyssal sediments accumulate oxide-scavenged water-column trace elements, with less lithogenic and small biogenic contributions. Post-depositional processes are not needed to explain metal enrichment in abyssal sediments: they probably only act to redistribute REE among the solid phases, for example, conversion from disordered to stable oxides^42^ and transfer from oxides to phosphates^15^, with limited impact on the sediment authigenic budget (see ‘REE carriers’ in Methods).

## Oxic diagenesis and benthic fluxes

Despite the efficient scavenging by settling particles, abyssal pore water is enriched in REE over bottom seawater, implying a diffusive flux to the ocean (Fig. 2). We use reactive-transport modelling to illustrate how oxic diagenesis explains the observed benthic fluxes (see ‘Diagenetic model’ in Methods).

**Extended Data Fig. 2:**
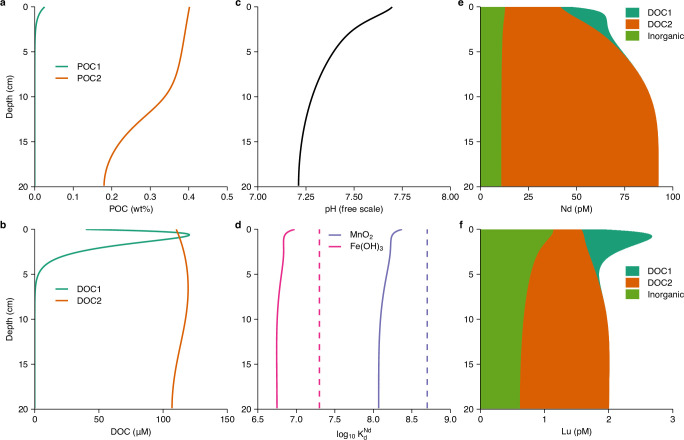
Modelling oxic diagenesis on the abyssal seafloor. **a**, Modelled particulate labile (POC1) and refractory (POC2) organic carbon fractions. **b**, Modelled dissolved labile (DOC1) and refractory (DOC2) organic carbon fractions. **c**, Modelled pore water pH. pH measured in unfiltered pore water extracted after centrifuge on board is 7.2 ~ 7.3 at Stns. 3 and 5, and 7.5 ~ 7.7 at Stn. 4. As noted in Methods, these data have finite utility given they are not measured in situ. **d**, Apparent K_d_ of Nd in oxides as controlled by oxide surface chemistry, pH and speciation (Methods, Diagenetic model). Dashed lines are the values derived from the water-column particle data (Methods: Particle scavenging). **e**, Modelled aqueous speciation of Nd, including complexation with the organic (DOC1 and DOC2) and inorganic ligands (mainly ${{{\rm{CO}}}_{3}}^{2-}$). **f**, Modelled speciation of Lu.

**Extended Data Fig. 3:**
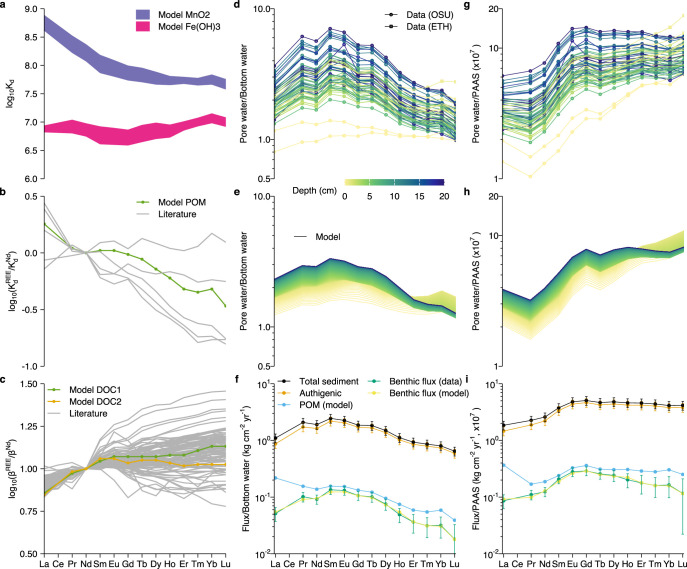
Modelling the pore water REE pattern. **a**, Apparent oxide *K*_*d*_ in the model (Methods: Diagenetic model). **b**, Apparent POM *K*_*d*_ in the model, relative to that of Nd and compared with literature data^93–95^. **c**, Stability constants of organic ligands in the model, relative to that of Nd and compared with literature data^126^. **d-e**, Measured and modelled REE concentrations normalized to the bottom water. **f**, Fluxes of the total solid, authigenic phases, POM-associated REE, and the benthic flux estimated based on the OSU data, compared with the modelled benthic flux. Error bars around the data estimates indicate 1σ uncertainties. **g-i**, Same as **d-f** but normalized to PAAS instead. Cerium is not included in the plots.

The apparent oxide *K*_d_ decreases from water column to sediment because POM remineralization lowers the pH and increases the organic ligand concentration in pore water (Extended Data Figs. 2 and 3). This change leads to net desorption of oxide-bound metals, despite the high absolute value of *K*_d_. POM-bound metals are also released, representing an amount that is small in the particulate flux but substantial for the porewater budget (Extended Data Figs. 2 and 3). In comparison, on continental margins, porewater pH is strongly buffered by alkalinity released during diagenesis, and kinetic reduction of oxides is the dominant source of benthic flux whereas equilibrium sorption is negligible^15^.

Our diagenetic model can reproduce the porewater-profile-based measurements of benthic flux (2.9 ± 0.5 pmol cm^−2^ yr^−1^ for Nd; ‘Benthic fluxes’ in Methods), which is smaller than the sinking particle flux, suggesting relatively low recycling efficiency (approximately 5%). The model also reproduces the porewater REE pattern (Extended Data Fig. 3): oxides and POM release a light REE (LREE)-enriched source to pore water, whereas preferential formation of organic complexes creates heavy REE (HREE) concentration peaks close to the interface (Fig. 2).

## Sedimentary imprint on the water column

Given the strong affinity of REE with oxides and the increase of oxide concentration with water depth (Fig. 1), we argue that scavenging constitutes a net removal from the water column. We tested this idea by creating a three-dimensional (3D) model of the Nd cycle using the transport matrix method^3,4,34^ (see ‘Water-column model’ in Methods). We use the ocean circulation inverse model^43^ with increased vertical resolution and state-of-the-art parameterization of bottom-intensified mixing^16^ (OCIM2-48L, 48 layers, 2° resolution). This model produces an improved representation of the abyssal Pacific circulation strongly shaped by seafloor topography^17,43^, allowing for a robust evaluation of the potential bottom-up control of the marine Nd cycle.

**Fig. 3:**
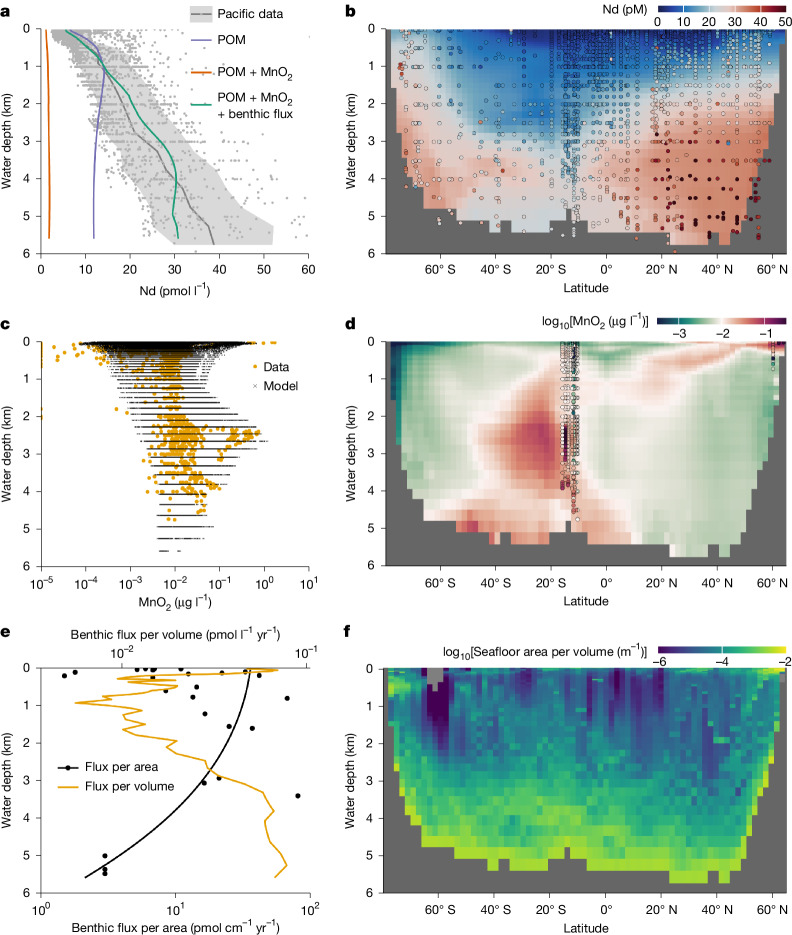
Seafloor imprint on water-column Nd concentration in the Pacific. **a**,**b**, Results of model experiments (see ‘Water-column model’ in Methods), including scavenging with: (1) POM only; (2) with added MnO_2_ scavenging; and (3) with added benthic flux. **a**, Modelled horizontally averaged concentrations of all three experiments. **b**, Modelled zonal mean concentrations for experiment (3) (see Extended Data Fig. 4 for scavenging by other biogenic particles, and Extended Data Fig. 5 for results of experiments (1) and (2)). Compiled Pacific seawater data are indicated by grey dots in **a** and colour-coded dots in **b** (see ‘Nd data compilation’ in Methods). The mean values and 1*σ* range of the data are shown by the grey line and shading, respectively, in **a**. **c**,**d**, Estimated particulate MnO_2_ concentrations (see ‘Water-column model’ in Methods; also see Extended Data Fig. 6). The GEOTRACES data^25,38^ are compared with neural-network-derived results on the model grids in **c**, and the zonally averaged concentrations (background colour) are shown in **d** together with GEOTRACES data (coloured dots). **e**,**f**, Benthic flux and the geometry of the ocean basin^57^, showing the depth distributions of benthic flux per unit area and unit volume (horizontal average; **e**) and seafloor-area-to-volume ratio (zonal average; **f**). In **e**, the black dots are porewater-based estimates of per unit area flux (see ‘Water-column model’ in Methods), the black line is its depth profile used in the model and the yellow line is the corresponding per unit volume flux. This flux profile is not made to best-fit the existing data, which is highly scarce, but to demonstrate that given reasonable estimates of benthic flux within the constraint of the data, we can explain the depth profiles of seawater Nd. In **f**, the area-to-volume ratio is computed using subgrid bathymetry based on ETOPO 2022^57^ (also see Extended Data Fig. 7).

**Extended Data Fig. 4:**
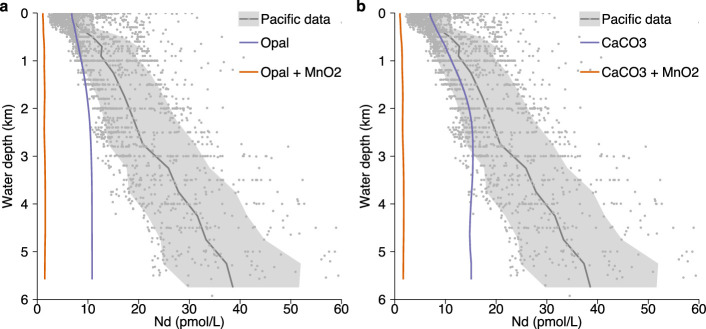
Scavenging of Nd by different biogenic particles in the Pacific. **a**, Modelled seawater Nd concentration considering (1) opal scavenging only and (2) further including MnO_2_ scavenging. **b**, Modelled seawater Nd concentration considering (1) CaCO_3_ scavenging only and (2) further including MnO_2_ scavenging. The results are plotted using zonally averaged concentrations. The compiled Pacific seawater data are indicated by grey dots. The mean values and 1σ range of the data are shown by the grey line and shade respectively. The model results of POM are shown in Fig. 3a.

**Extended Data Fig. 5:**
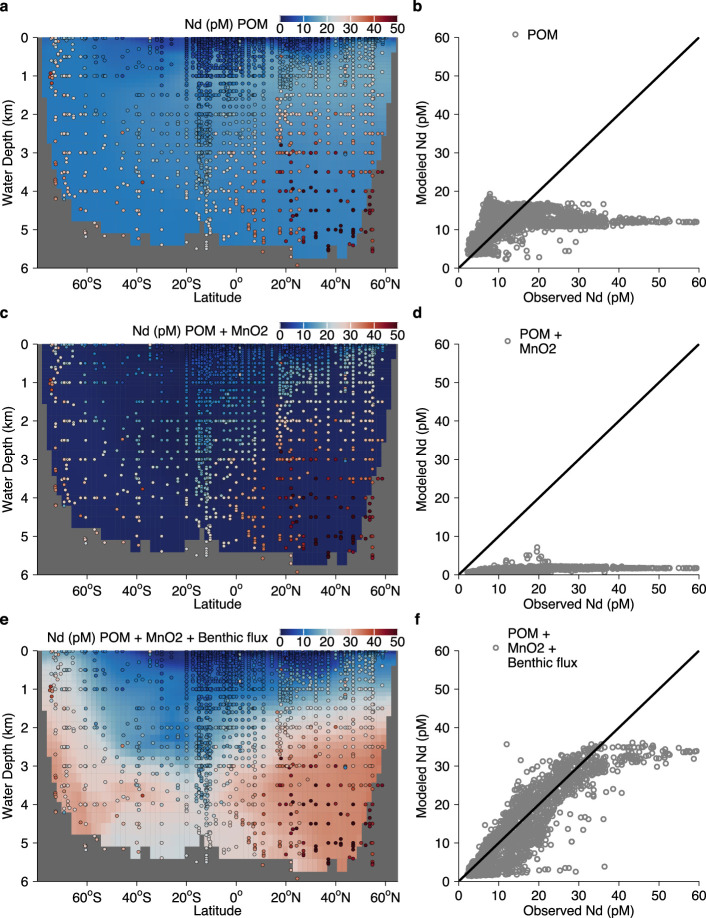
Impact of particle scavenging and benthic flux on modelled seawater Nd concentration in the Pacific. **a-b**, Model experiment only including scavenging by POM. Observations of Nd concentration are indicated by coloured dots, and modelled zonal average Nd concentration are shown as the background colour in **a**. Observed versus modelled Nd concentrations is show in **b**. **c**-**d**, same as **a**-**b**, but showing model experiment further including MnO_2_ scavenging. **e**-**f**, same as **c**-**d**, but showing model experiment further adding benthic flux. The horizontally averaged Nd concentration profiles of these experiments are shown in Fig. 3a.

Supplying only river and dust fluxes to the model, we reproduce the classic behaviour of metals in reversible scavenging OBMs^3–5,13^: the dissolved element concentration increases with water depth as biogenic particles shuttle surface sources downwards (Fig. 3a and Extended Data Figs. 4 and 5). Here we use POM as the only biogenic scavenger, suggested by our analysis of the GEOTRACES data. The model can fit the Nd concentration profile in the upper water column only at the expense of underestimating the deeper Nd concentrations, making the profile more nutrient-like than the nearly linear observations. This OBM disparity issue is often overcome in the literature by specifying carbonate and opal as scavengers of trace elements^3,13^, taking advantage of their longer remineralization length scales. We can reproduce similar results by allowing the *K*_d_ of these biogenic particles to be free tuning parameters (Extended Data Fig. 4), but our analysis of GEOTRACES data (Fig. 1) shows that such model exercises are not consistent with observations.

**Extended Data Fig. 6:**
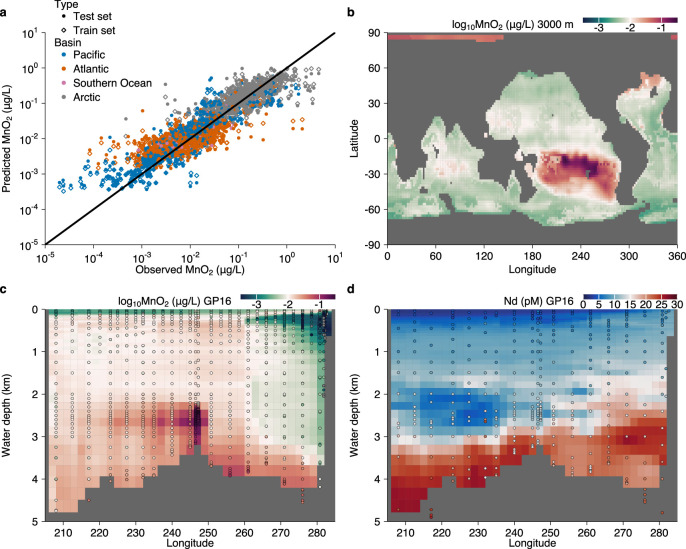
Distribution of MnO_2_ in the ocean generated using artificial neural network (ANN, see Methods: Water-column model). **a**, MnO_2_ concentration predicted by ANN compared with the GEOTRACES observations^38^, showing both the training and testing datasets (both are 500-member ensembles). **b**, Predicted MnO_2_ concentration at 3000 m depth. **c**, Predicted MnO_2_ concentration at the locations of GEOTRACES GP16 cruise compared with observations^25,38^ (coloured-dots). The ANN prediction captures localized features including oxygen minimum zone, hydrothermal plume and benthic nepheloid layers. **d**, Modelled Nd concentrations at the locations of GEOTRACES GP16 cruise compared with observations^23,38,61^ (coloured-dots). Note that scavenging by high MnO_2_ concentration in hydrothermal plume and benthic nepheloid layers reduces modelled Nd concentration.

To implement oxide scavenging, we generate a 3D Mn oxide concentration field by training an artificial neural network using GEOTRACES data^25,38^ (Extended Data Fig. 6 and ‘Water-column model’ in Methods). Once we add Mn oxide to the model, scavenging becomes a net sink of Nd throughout the water column (Fig. 3a–d and Extended Data Figs. 4 and 5). Although scavenging is implemented as a reversible mechanism, the net result appears as an irreversible process. This result illustrates that, for metals with an affinity for Mn oxide much greater than for biogenic particles, their increasing concentrations with water depth requires either an internal (for example, Th) or a boundary source (for example, REE).

We propose that benthic fluxes, originating mostly from the abyssal seafloor, explain the water-column REE concentration increase for two reasons.

**Extended Data Fig. 7:**
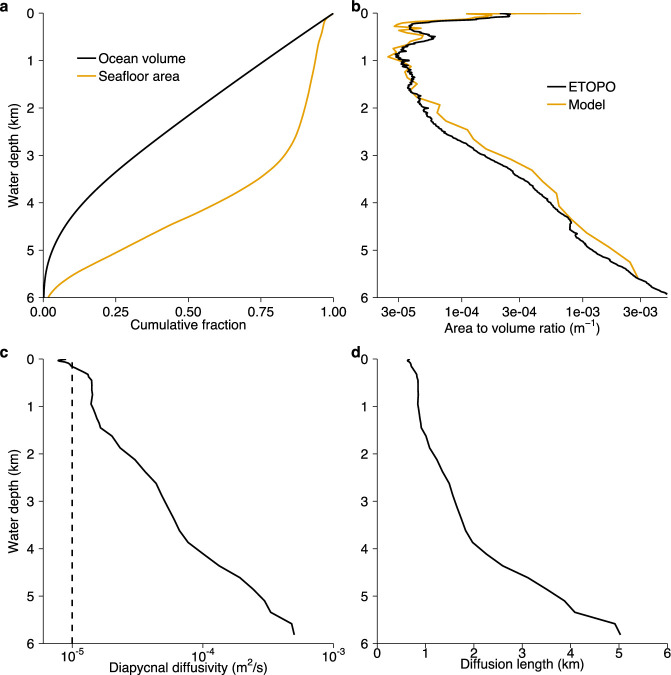
Seafloor geometry and bottom-intensified mixing in the Pacific. **a**, Distributions of ocean volume and seafloor area respectively, showing the change of cumulative fractions with depth, starting from the bottom-most. Results are computed using the ETOPO 2022 bathymetry^57^. **b**, Distribution of seafloor area to ocean volume ratio with depth, in the ETOPO 2022 bathymetry^57^ and the derived sub-grid bathymetry for OCIM2-48L^34^ respectively. **c**, Estimated bottom-intensified turbulent mixing^16^, manifested as increasing diapycnal diffusivity (horizonal average) with water depth. **d**, The corresponding diffusion length of Nd.

First, the shape of ocean basins is such that the abyssal ocean has the most seafloor area but only a fraction of the ocean volume, resulting in an exponential increase of the area-to-volume ratio with water depth (Fig. 3e,f and Extended Data Fig. 7a,b). Although the continental margins have much higher benthic flux per unit area^44^, the abyssal seafloor dominates the total sedimentary flux because of its areal predominance (Fig. 3e). Consequently, in the abyssal ocean, even a small benthic flux per unit area has a large impact on metal concentration per unit volume.

Second, diapycnal diffusivity increases exponentially from about 10^−5^ m^2^ s^−1^ in the upper ocean to about 10^−3^ m^2^ s^−1^ in the abyss because of the turbulence generated by internal tides impinging on seafloor topography^16^ (Extended Data Fig. 7c and ‘Bottom-intensified mixing’ in Methods). Accordingly, the diffusion length of Nd increases with depth from about 1 km to about 5 km (Extended Data Fig. 7d), implying a large vertical impact of abyssal benthic sources.

Adding a benthic flux to our model successfully simulates the first-order feature that Nd concentration increases nearly linearly with depth in the Pacific (Fig. 3a,b, Extended Data Fig. 5 and ‘Water-column model’ in Methods). Superimposed on this large-scale distribution, our model also captures the spatial heterogeneity owing to localized factors such as hydrothermal plumes and nepheloid layers near the East Pacific Rise (Fig. 3 and Extended Data Fig. 6). The model underestimates abyssal Pacific Nd concentrations near 40° N, where a previous study used radium (Ra) isotopes to infer anomalously high benthic flux and/or sluggish mixing^45^. Such regional features are not included in our large-scale model. Overall, our model clearly illustrates how the dynamic interplay between scavenging and benthic source controls the water-column metal distribution, seen clearly in the Nd cycle owing to its moderate residence time (466 years in this model).

## Recycled versus ‘new’ benthic fluxes

**Fig. 4:**
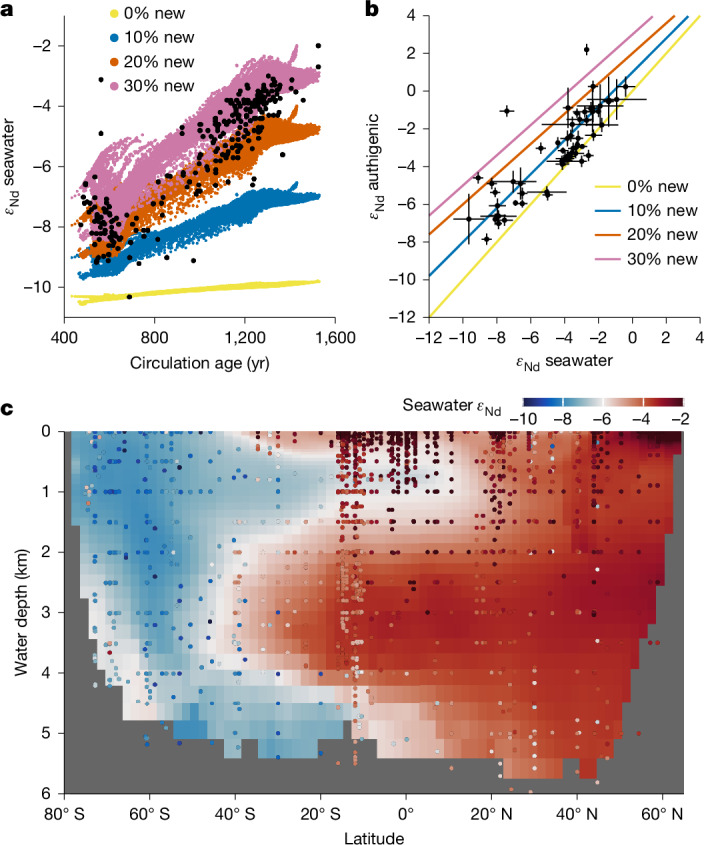
Seafloor imprint on water-column *ε*_Nd_ in the Pacific. **a**, Abyssal (>3,000 m) ocean *ε*_Nd_ is altered towards more radiogenic (that is, positive) values with increasing water-mass age, because of prolonged exposure to a new benthic flux driven by weathering of sedimentary volcanic silicates. The black dots indicate seawater data and the coloured dots indicate the model grid. The colour refers to the model experiment, in which the new source accounts for 0% to 30% of the total benthic flux (see ‘Water-column model’ in Methods). In these experiments, the new source is added to all seafloor areas in the Pacific. Extended Data Fig. 10 shows experiments of adding the new source to various subregions of the seafloor. **b**, Relationship between the *ε*_Nd_ of authigenic sediment and overlying seawater, indicating that new sources are needed to explain the authigenic data. Paired observations are binned into ocean boxes and indicated by black dots with standard deviations (see ‘Nd data compilation’ in Methods and Extended Data Fig. 8); the coloured lines indicate mixing between seawater and 0% to 30% of new benthic source. **c**, Modelled zonally averaged seawater *ε*_Nd_ in the experiment where we add 30% new source to all Pacific seafloor areas (see Extended Data Fig. 9 for experiments with approximately 0–20% new source). Compiled Pacific data are indicated by coloured dots.

**Extended Data Fig. 8:**
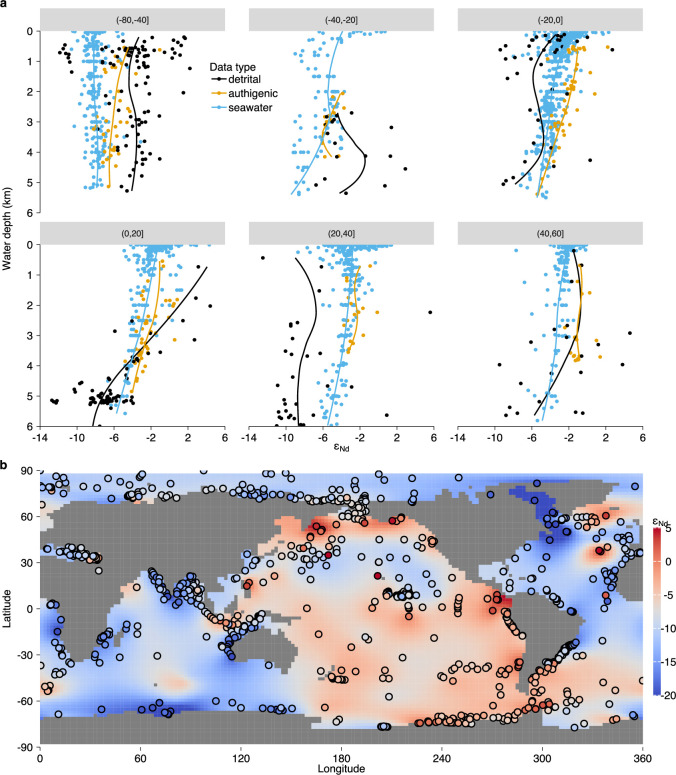
Distributions of ε_Nd_ in the Pacific (Methods: Nd data compilation). **a**, Latitudinal evolution of detrital sediment, seawater and authigenic ε_Nd_ in the Pacific. Data are binned according to their latitude (bin range indicated on top) and presented from south to north. Dots indicate the original data, and lines are the LOESS results. **b**, Spatial distribution of core-top detrital sediment ε_Nd_. Data dots are superimposed on the interpolated field created using variational analysis^127^. Note the North Pacific dust province indicated by low ε_Nd_ values (less than −8 as used in the sensitivity experiments in Extended Data Fig. 10). This compilation is an update of that in ref. ^9^.

The results above demonstrate the potentially dominant control of boundary exchange on water-column concentration profiles. Here we further show that the abyssal benthic flux is capable of transforming water-column isotope distributions on large scales. In the classic top-down-only view^3,35^, water-mass endmembers acquire their *ε*_Nd_ signatures at the surface ocean, and in the interior seawater, the *ε*_Nd_ distribution reflects conservative mixing of these endmembers. This view is inconsistent with the observation that abyssal Pacific *ε*_Nd_ is non-conservative, as shown by the strong correlation of *ε*_Nd_ with water-mass age^9,36^ (Fig. 4a). There is no deep-water formation in the North Pacific, and the predominant deep-water source is from the well-mixed Southern Ocean, which has *ε*_Nd_ of about −9 *ε* (Extended Data Fig. 8 and ‘Nd data compilation’ in Methods). As deep water traverses northwards, *ε*_Nd_ becomes increasingly radiogenic (more positive *ε*_Nd_ values), reaching >−4 *ε* in the North Pacific (Fig. 4a). Reversible scavenging has been argued to bring radiogenic surface water signatures to the deep ocean^3^, but OBMs have struggled to simulate the observed *ε*_Nd_ of Pacific Deep Water^13,46^.

We adopt concepts from nutrient cycling, and partition the benthic flux into a recycled component owing to the regeneration of water-column scavenged Nd, and a newly introduced (‘new’) component arising from in situ marine silicate weathering^6,8,12,14,15,33,47^. Surface water *ε*_Nd_ in the study region is radiogenic (about −3 *ε* to 0 *ε*; Extended Data Fig. 8), whereas porewater and bottom-water *ε*_Nd_ are similar at about −4.5 *ε* (Fig. 2). This similarity indicates that the abyssal benthic flux in the study region is mainly recycled, and regenerated Nd is primarily scavenged from the deep ocean. Following the water-column cycling model above, such a recycled flux readily explains the non-conservative increase of Nd concentration, but it has little power to alter abyssal *ε*_Nd_.

**Extended Data Fig. 9:**
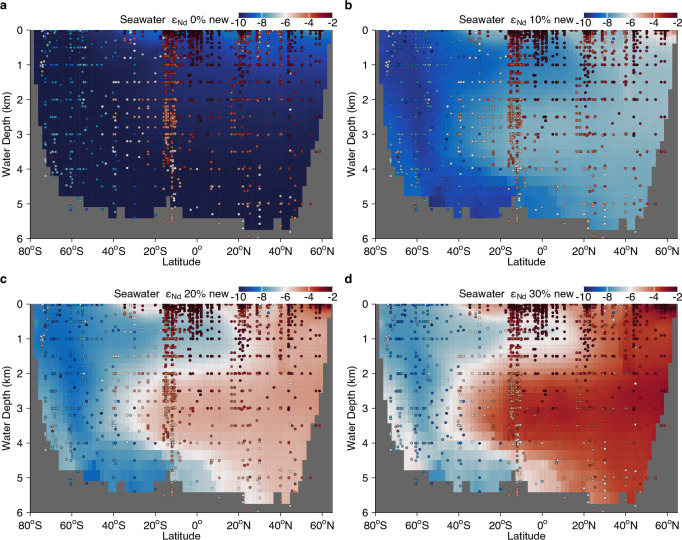
Impact of the new benthic source on modelled seawater ε_Nd_ in the Pacific. **a**, Model experiment without any new volcanic benthic source. Observations are marked by coloured dots, and modelled zonally averaged seawater ε_Nd_ in the Pacific are shown in the background colour. **b**-**d**, same as **a**, but with the new source accounting for 10%, 20%, and 30% of the total benthic source respectively. In these experiments, the new source is supplied from all seafloor areas in the Pacific. See Extended Data Fig. 10 for model experiments of adding the new source to sub-regions of the seafloor.

In contrast, a new benthic flux derived from weathering of volcanic silicates (*ε*_Nd_ ≈ +10) sourced from the Pacific Ring of Fire would explain the non-conservativity of *ε*_Nd_ (refs. ^6,8,15,47^). We tested this idea by incorporating Nd isotopes in the water-column model (see ‘Water-column model’ in Methods). Our experiments show that, without a new source, reversible scavenging has limited power to explain the radiogenic nature of Pacific Deep Water, and the abyssal Pacific simply inherits the *ε*_Nd_ of its Southern Ocean source waters (Fig. 4a and Extended Data Fig. 9). In contrast, with a new component constituting 10% to 30% of the total benthic flux added to all seafloor areas in the Pacific, prolonged exposure to this flux owing to weak meridional circulation transforms the abyssal Pacific *ε*_Nd_ towards observed radiogenic values (Fig. 4a,c and Extended Data Fig. 9). Sediment studies are consistent with this partition of benthic flux. In the Northeast Pacific, the only other location with porewater *ε*_Nd_ data, benthic fluxes are more radiogenic than bottom water, allowing for up to 20% new component in the larger benthic fluxes found there^33^. Authigenic sediments in the Pacific are also consistently more radiogenic than bottom water^9^; if these authigenic data mirror porewater *ε*_Nd_ (refs. ^33,47^), they too are consistent with a 10% to 30% new benthic flux component (Fig. 4b). In comparison, our model results show that even greater proportions of new benthic flux is needed to explain the highly radiogenic *ε*_Nd_ of intermediate waters (Fig. 4c), for example in the equatorial western Pacific where intense boundary exchange with basaltic islands was discovered^6^.

**Extended Data Fig. 10:**
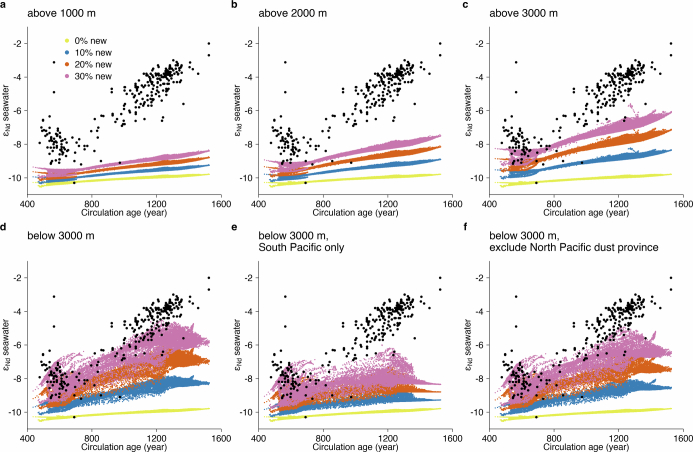
Sensitivity of modelled abyssal (below 3000 m) Pacific seawater ε_Nd_ to the location where the new benthic source is supplied. **a-c**, Results of supplying the new source only from the margins, up to water depths of 1000 m, 2000 m and 3000 m respectively. **d-f**, Results of supplying the new source only from seafloor below 3000 m, either everywhere (**d**), or only in the South Pacific (**e**), or everywhere except the North Pacific dust province (**f**) where detrital sediment is dominated by Asian dust (see Extended Data Fig. 8). Black dots indicate seawater data, and coloured dots indicate model grid. The colour refers to the model experiment, in which the new source account for 0% to 30% of the total benthic flux. These experiments are compared with the one in Fig. 4a and Extended Data Fig. 9, where the new source is supplied from all Pacific seafloor areas.

Model sensitivity experiments further show that abyssal seawater *ε*_Nd_ cannot be explained if boundary exchange is restricted to the margins (see ‘Water-column model’ in Methods). Limiting the new source to the margins, the model can explain only about +2 *ε* out of the roughly +6 *ε* increase of abyssal *ε*_Nd_ from the Southern Ocean to the North Pacific. The rest must be attributed to new benthic flux from the abyssal seafloor (Extended Data Fig. 10). In the Pacific, this new source depends on the availability of reactive volcanic materials^6,8,15,47^. The lack of a new source at our study sites in particular is consistent with the fact that the dominant local detritus is refractory Asian dust (*ε*_Nd_ = –10)^48^ (Extended Data Fig. 8). Comparing compiled seawater, authigenic and detrital data^9^, we suggest that the addition of new abyssal sources occurs in the South Pacific, and the North Pacific outside the dust province, where there are considerable volcanic inputs to abyssal sediments (Extended Data Fig. 8). Our model tests support this suggestion (Extended Data Fig. 10).

Thus, the benthic flux is composed of a mainly recycled flux, and a minor new source that has an *ε*_Nd_ that is sharply different from the source water mass in the Pacific. The proportion of new source is large enough to prevent the water-mass *ε*_Nd_ from being conservative, but sufficiently small that seawater *ε*_Nd_ is still impacted by ocean circulation and mixing without being overwhelmed by local sedimentary processes.

## An integrated framework

**Fig. 5:**
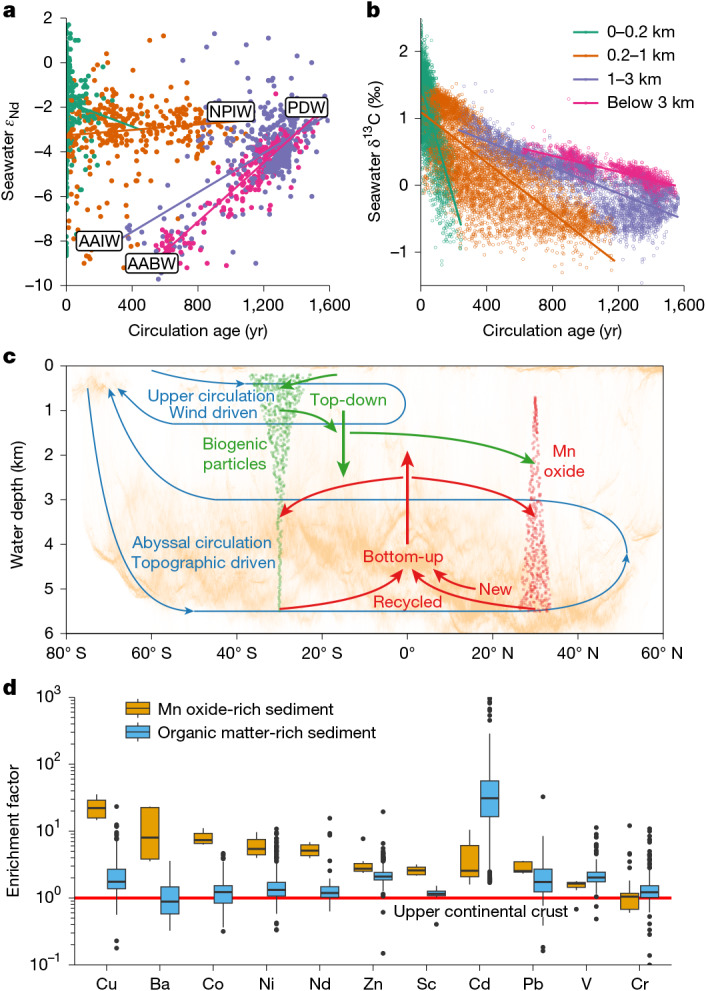
The bottom-up and top-down biogeochemical cycles. **a**,**b**, Comparing the bottom-up driven tracer *ε*_Nd_ (**a**) with the top-down driven tracer δ^13^C (ref. ^58^; **b**). The non-conservativity of *ε*_Nd_ is higher in the abyssal ocean whereas for δ^13^C it is the greatest at the surface ocean. Compiled seawater data are colour-coded by the depth range. Major Pacific water masses are shown in the plot, including Antarctic bottom water (AABW), Antarctic intermediate water (AAIW), Pacific deep water (PDW) and North Pacific intermediate water (NPIW). **c**, Conceptual model of bottom-up versus top-down biogeochemical cycles. The background colour is the distribution of the seafloor area in ETOPO 2022^57^. The cycling of a top-down tracer mainly consists of surface uptake and regeneration in the upper ocean. The cycling of a bottom-up tracer is dominated by benthic processing that largely happens on the oxic abyssal seafloor. The top-down and bottom-up processes are linked by particle production and scavenging. The affinity to biogenic versus oxide particles controls the relative importance of top-down and bottom-up processes in a tracer’s distribution. A tracer’s biogeochemical cycle is strongly tied to ocean circulation, which controls the preformed properties and dictates the exposure time to water-column and benthic processing. **d**, Affinity of selected trace elements to authigenic oxides compared with that to organic matter. The affinity is shown using elemental enrichment factors in oxide-rich sediments (this study) and organic-rich sediments^49^ (greater than 2 wt% organic carbon content), calculated as metal-to-aluminium-concentration ratio in sediment relative to that in the upper continental crust^41^. The red line indicates no enrichment. In the boxplot, boxes show the interquartile range; thick lines show the median; whiskers extend to 1.5 × interquartile range away from the boxes; dots are outliers.

Our analysis of the marine REE cycles reveals an integrated mode of trace-metal cycling that is mainly driven by bottom-up processes such as oxic diagenesis and bottom-intensified turbulent mixing, but also coupled to top-down particle sinking and scavenging. The contrast with the top-down-only view is best illustrated by comparing the distributions of seawater δ^13^C with *ε*_Nd_ (Fig. 5a–c). The change of δ^13^C with water-mass age becomes weaker and its non-conservativity decreases with increasing water depth, as organic carbon cycling mainly happens in the upper ocean. This contrasts with the age-dependent *ε*_Nd_ transformation, as Nd recycling is focused on the abyssal seafloor (Fig. 5a–c). Thus, the source and mode of redistribution in the oceans (for example, remineralization versus scavenging and water-column versus sediment processing) is critical to understanding and interpreting the tracer function (for example, as tracers of circulation).

Our model further highlights the importance of particle-type-dependent scavenging. How strongly a trace metal is partitioned into oxides versus biogenic particles is a key control over its distribution. We qualitatively evaluate this relative affinity by comparing the enrichment factors of trace metals in oxide-rich abyssal sediments with those in organic-rich sediments^49^ (Fig. 5d). Contrary to REE, for example, cadmium (Cd) appears to represent an endmember that strongly favours association with organic matter, consistent with its water-column profile being the most nutrient-like among trace elements^21^. Copper, colbalt, nickel and zinc are bio-essential, but they also have a high affinity for Mn oxide. Accordingly, elements like Cu should have scavenged-type water-column profiles, but are actually observed to increase linearly^50^, like Nd. Our framework thus predicts that there is strong benthic flux of Cu from abyssal sediment, a prediction supported by observations and models^19,20,31^.

We argue that the benthic impact is widespread among trace metals based on existing porewater data from the abyssal seafloor^19,20^, and draw attention to some key implications for using TEIs in tracing ocean processes.

First, marine silicate weathering can provide a new source of trace elements and thus may explain disparities in isotopic budgets of some marine metals^14^. For Fe and Nd, this appears to be a robust conclusion^6,8,15,28^, and similar arguments have been made for Cu (ref. ^50^). Beyond a better understanding of tracers, marine silicate weathering may consume as much carbon dioxide as continental silicate weathering^51^. Our model provides a foundation to re-evaluate the role of silicate weathering, no longer a strictly continental phenomenon^52,53^, as a buffering agent of climate change and a driver of ocean biogeochemical evolution.

Second, deep-ocean processes, such as hydrothermalism and oxygenation, affect the global ocean budgets of trace elements. For example, hydrothermalism today is the dominant source of both dissolved seawater Mn and thus of particulate Mn oxide^54^ (Fig. 3c,d and Extended Data Fig. 6). Our model shows that variations in Mn-oxide cycling will exert strong control over trace-metal behaviours (Fig. 3b), even those that are not traditionally seen as sensitive to hydrothermalism and redox^55^. Our model thus offers a different way of understanding and interpreting a wide range of ocean tracers with respect to hydrothermalism, ventilation and the redox nature of the deep sea.

Finally, our model places emphasis on the geometry of the ocean basins via its impact on abyssal mixing and circulation^17^. For example, today, bottom-intensified mixing is concentrated near mid-ocean ridges^17^, and thus the average depth of the crest (about 2.5 km) delineates the vertical reach of the abyssal sedimentary imprint (Fig. 5c). Few, if any, OBMs have investigated the biogeochemical impact of topographic mixing. We argue that the evolution of palaeobathymetry will be important in better understanding of the long-term history of ocean biogeochemistry.

## Methods

### Particle scavenging

We quantitatively partitioned scavenged Nd into fractions associated with each particle type. First, we estimated the particle-specific partition coefficients of Nd. Assuming reversible scavenging, the concentration of scavenged Nd (Nd_s_) is:

1 $${{\rm{Nd}}}_{{\rm{s}}}=\sum _{{\rm{i}}}{K}_{{\rm{d}}}^{i}\times {C}^{i}\times {{\rm{Nd}}}_{{\rm{d}}},$$

where *i* is the index of particle type, including Mn oxide, Fe oxide, POM, calcium carbonate (CaCO_3_), opal and lithogenic particle, ${K}_{{\rm{d}}}^{i}$ and *C*^*i*^ are the apparent partition coefficient and particle concentration of the *i*th particle type, and Nd_d_ is the total dissolved Nd concentration. Here we assume that ${K}_{{\rm{d}}}^{i}$ is a constant, a standard practice in the literature^24,39^. When using the total dissolved concentration in equation (1), ${K}_{{\rm{d}}}^{i}$ is an apparent constant, as in principle it should depend on metal speciation^59^. Given the relative uniformity of deep-ocean properties, it is reasonable to assume that speciation in seawater is roughly the same and treat ${K}_{{\rm{d}}}^{i}$ as a constant. However, in pore water, speciation can strongly differ from seawater and changes with sediment depth; it is thus preferable to model ${K}_{{\rm{d}}}^{i}$ as a variable (see below).

Equation (1) is rewritten following refs. ^24,39^:

2 $${K}_{{\rm{d}}}^{{\rm{TP}}}=1/{\rm{TP}}\times {{\rm{Nd}}}_{{\rm{s}}}/{{\rm{Nd}}}_{{\rm{d}}}=\sum _{{\rm{i}}}{K}_{{\rm{d}}}^{i}\times {f}^{i},$$

where TP is the total particle concentration, ${K}_{{\rm{d}}}^{{\rm{TP}}}$ is the total particle partition coefficient and *f*^*i*^ is the mass fraction of the *i*th particle type.

We used data from the GEOTRACES Pacific GP16 and Atlantic GA03 cruises to characterize the particle scavenging of Nd (refs. ^25,38,60,61^). These are so far the only cruises reporting both dissolved and particulate Nd data, as well as necessary bulk particulate data to carry out this analysis. The particulate data were size-fractionated (small particle, about 1–51 μm; large particle, >51 μm). Here we assume that *K*_d_ is not size specific. The GEOTRACES dissolved and particle samples were collected using Niskin bottles and McLane pumps, respectively, and thus are from slightly different depths. To create a paired dataset, we interpolated the dissolved Nd concentration at the depths of the particulate data.

The concentration of scavenged Nd is calculated by removing the lithogenic background from the measured total particle Nd concentration. In the original studies^25,60^, titanium (Ti) and aluminium (Al) were used for detrital correction of the Atlantic GA03 and Pacific GP16 data, respectively, to estimate the concentrations of lithogenic particle and authigenic Fe oxide and Mn oxide. To be consistent, we also used Ti and Al for detrital correction of Nd at the GA03 and GP16 sites, respectively, assuming that the detrital Nd/Ti or Nd/Al ratio is the same as the upper continental crust^41^.

Equation (2) forms a system of equations that we solved for ${K}_{{\rm{d}}}^{i}$ using least-square estimation. To ensure the non-negativity of ${K}_{{\rm{d}}}^{i}$, the estimation was performed in terms of ${\log }_{10}{K}_{{\rm{d}}}^{i}$. We used a global black-box optimization method with constraints (ranges of ${\log }_{10}{K}_{{\rm{d}}}^{i}$ were set to −10 to 10) from the Julia package BlackBoxOptim.jl^62^. To account for data heterogeneity and provide error estimates, we performed the estimation 1,000 times by randomly sampling 75% of the data each time. When all particle data were used, the estimation gave ${\log }_{10}{K}_{{\rm{d}}}^{i}$ values of 8.66 (median, 8.63 to 8.72 interquartile range), 7.21 (7.17 to 7.26), 5.55 (5.10 to 5.81), 6.12 (5.92 to 6.22), −2.33 (−9.53 to 5.38) and −9.53 (−9.79 to −9.24) for Mn oxide, Fe oxide, POM and CaCO_3_, lithogenic particles and opal, respectively. This shows that lithogenic particles and opal are negligible scavengers of Nd, consistent with the observation that ${K}_{{\rm{d}}}^{{\rm{TP}}}$ has no statistically significant (*P* > 0.1) relationship with lithogenic particles and opal *f*^*i*^. However, if we restrict the particle data to below the surface (>200 m), our estimate shows that ${\log }_{10}{K}_{{\rm{d}}}^{i}$ of Mn oxide, Fe oxide and POM still have narrowly defined distributions overlapping with the aforementioned estimates, but ${\log }_{10}{K}_{{\rm{d}}}^{i}$ of CaCO_3_ has a bimodal distribution with a wide interquartile range of −8.93 to 6.07 (median of 5.74), consistent with the observation that the correlation of ${K}_{{\rm{d}}}^{{\rm{TP}}}$ with ${f}^{{{\rm{CaCO}}}_{3}}$ is statistically insignificant (*P* > 0.1) below the surface. This result shows that CaCO_3_ may be an important scavenger of Nd in the surface ocean but not at depth. Alternatively, this disparity may be the result of neglecting aqueous speciation when estimating ${K}_{{\rm{d}}}^{i}$, considering that seawater Nd exists mainly as carbonate complex and the carbonate speciation differs between the surface and deep^59^. We are mainly interested in particle scavenging beneath the surface. Thus, we ignore the contribution of CaCO_3_ to Nd scavenging in Fig. 1. However, to be comparable with previous reversible scavenging models, we included model sensitivity experiments where we allowed scavenging onto opal and CaCO_3_, as discussed later. Finally, we performed an estimation including only oxides and POM. The estimated ${\log }_{10}{K}_{{\rm{d}}}^{i}$ and associated uncertainty of this final analysis are reported in the main text.

Our result of the varying affinity of Nd with particle types are consistent with previous field and laboratory studies. Chemical extractions of the labile proportion of marine particles have REE patterns that resemble that of the Mn oxide^11,63^. Aqueous and surface complexation modelling have suggested that in the deep ocean, the dissolved seawater REE patterns are most likely explained by scavenging onto Mn oxide^59^. In contrast, it has long been known that pristine biogenic carbonate contains little REE^64^, and the REE associated with planktic foraminifera are predominantly hosted by oxides attached to the shell rather than within biogenic carbonate^65^. Moreover, sediment-trap studies have found that settling biogenic opal does not contain a resolvable amount of LREE (although it may contain some HREE)^66^. The evidence all points to Mn oxide as the dominant REE scavenger, particularly in the deep ocean, whereas the roles of biogenic carriers, especially carbonate and opal, are negligible.

### Pacific cruise

We collected water-column, sediment and porewater samples during the KM2012 cruise (October 2020) onboard R/V *Kilo Moana* at 3 sites from the equatorial North Pacific along 152° W (Station 3, 11° N, 5,388 m; Station 4, 3° N, 5,050 m; Station 5, 7° N, 5,502 m). Sampling procedures followed ref. ^44^ and are briefly summarized here. Water-column samples were collected and filtered (<0.45 μm) from Niskin bottles on a CTD Rosette. Sediment cores were collected using an MC800 multi-corer and sectioned at 1.2-cm intervals. Porewater aliquots for REE concentration and *ε*_Nd_ were collected by centrifuge and filtration (<0.45 μm). The REE aliquots were extracted from one core, and the *ε*_Nd_ aliquots combined extractions from corresponding layers of 20 to 25 cores. The REE aliquots include porewater samples from the top 20 cm and all seawater samples from each station, whereas the *ε*_Nd_ aliquots are fewer, having only porewater samples from the top 10 cm and the 3 bottom-most seawater samples at each station. Porewater aliquots for nutrient analyses were extracted using Rhizon samplers and filtered (<0.2 µm). Porewater aliquots for dissolved organic carbon (DOC) analysis were extracted via Rhizon, filtered (<0.45 μm with GFF filter), and poisoned with mercuric chloride and stored in a glass vial. Porewater dissolved oxygen (O_2_) concentration was measured using a fibre-optic oxygen microsensor inserted into pre-drilled holes through the core into the sediments. The sensor readings were converted to O_2_ concentration by linearly calibrating against the published bottom-water O_2_ concentration at the study sites. Porewater pH was measured on unfiltered samples using a portable pH sensor post-extraction via centrifugation. Analysis of pH in pore waters is notoriously difficult owing to the changes in pressure upon recovery, and thus the pH data have finite utility. Sediment samples were frozen onboard and freeze-dried in the lab.

Elemental concentrations of the REE aliquots were analysed at Oregon State University using an Elemental Scientific seaFAST-pico offline pre-concentration technique, and the procedure has been extensively documented as part of the GEOTRACES intercalibration effort^67^. Elemental concentrations of the *ε*_Nd_ aliquots (approximately 10 ml) were analysed at ETH Zurich using Nobias Chelate-PA1 resin in a manual column procedure^68^. The measured REE concentration and pattern from the two labs agree well (Fig. 2 and Extended Data Fig. 3), and the small differences are expected given that the REE and *ε*_Nd_ aliquots represent samples extracted from different cores. The porewater REE concentration of the REE aliquots measured at Oregon State University shows a deep peak between 10 cm and 15 cm, but the scatter in observations of this peak is large among the 3 sites (Fig. 2), despite similarities in the REE pattern between all samples analysed (Extended Data Fig. 3). Unfortunately, the *ε*_Nd_ aliquots did not extend to this interval and thus we were not able to confirm this feature at ETH Zurich. While inherently interesting, this deeper peak does not affect our estimation of the benthic flux, which depends on only the REE concentration data at the sediment–water interface. Thus, for the intention of this paper, we have omitted interpretation of this feature. Also, we do not discuss cerium (Ce) in this study, because of the large inter-lab disagreement shown in the GEOTRACES intercalibration effort^67^, and its fractionation from other REE owing to redox sensitivity.

Isotope analysis of the *ε*_Nd_ aliquots (about 400–700 ml of pore water or about 1.5 l of seawater) were done at ETH Zurich. Samples were buffered to a pH of 5.5 ± 5 and pre-concentrated using an in-house extraction manifold containing Nobias Chelate-PA1 resin. The procedure has been extensively used for trace-metal isotope analysis at ETH Zurich^69,70^. After pre-concentration, separation of Nd from the matrix elements and other REE was done using Eichrom RE and LN spec resins. Ultimately, about 5–10 ng of Nd was available for isotope measurements. Procedural blanks were <3 pg. Isotope analysis was done on a Neptune Plus multi-collector inductively coupled plasma mass spectrometer (Thermo Fisher) following the procedure of ref. ^71^. Internally normalized sample data were renormalized to the ^143^Nd/^144^Nd ratio of La Jolla^72^. Repeated analysis of 8-ppb La Jolla solutions results in a long-term external reproducibility of 0.27 *ε* (2*σ*). Nd isotope analysis was also quality-controlled by repeated measurements of the US Geological Survey reference materials BCR-2 (*ε*_Nd_ = −0.11 ± 0.25, 2*σ*) and BHVO-2 (*ε*_Nd_ = 6.70 ± 0.24, 2*σ*) at the same concentration (about 5–10 ppb) as the porewater samples in agreement with literature results^73^.

Nutrients were analysed at Oregon State University using a Technicon AutoAnalyzer II (phosphate and ammonium) and an Alpkem RFA 300 (silicic acid, nitrate + nitrite). The method and data processing follow ref. ^74^. DOC was analysed with a V-CSN/TNM-1 (Shimadzu) at the Scripps Institution of Oceanography following ref. ^75^. Sediment samples were analysed for total organic carbon contents using a GVI (now Elementar) Isoprime 1000 with Eurovector EA at Bigelow Laboratory for Ocean Sciences. Samples were measured for the total carbon (organic plus inorganic) and a separate sample split was acidified to remove carbonate and then measured for the organic fraction. Freeze-dried bulk sediments were digested in a concentrated HCl–HNO_3_–HF mixture at high pressure, using a microwave-assisted system (CEM MARS-6), and sediment elemental concentrations were measured using inductively coupled plasma optical emission spectroscopy and an inductively coupled plasma mass spectrometer at Oregon State University^76^. X-ray diffraction of freeze-dried raw samples were made at K/T GeoServices, using a Siemens D500 automated powder diffractometer equipped with a Cu X-ray source (40 kV, 30 mA) and a scintillation X-ray detector. Semi-quantitative determinations of whole-sediment mineral amounts were done using Jade Software (Materials Data) with the Whole Pattern Fitting option. Estimation of sedimentation rate was done by radiocarbon dating planktic foraminiferal shells from Station 4 (W. M. Keck Carbon Cycle AMS Facility, UC Irvine), where enough carbonate is available.

### Abyssal Pacific sediment

Save for the presence of considerable biogenic carbonates at Station 4 (30% to 73% calcite based on X-ray diffraction) compared with Station 5 (0%) and Station 3 (about 0–1.6%), the sediment mineralogy is similar at all sites, being composed of quartz (about 4–11%), plagioclase (about 3–10%) and illite + mica (about 6–15%), with lesser components of K-feldspar (about 0.7–4%) and chlorite (about 0.7–3%), assuming halite and gypsum reflect dried pore water. There is a large fraction of a semi-quantifiable amorphous phase at all sites (about 40%).

Dissolved oxygen and nitrate + nitrite indicate that aerobic respiration dominates organic-matter remineralization and that diffusion from seawater is sufficient to keep the sediment package oxygenated with no sign of denitrification or more reducing conditions within the upper 20 cm (Fig. 2). Consequently, porewater Fe and Mn was below our detection limits (0.1 μM) in all cores. This diagenetic setting is consistent with previous work in the area^77^. The relatively low organic-matter rain rate^77^ (about 0.1–0.4 mmol m^−2^ d^−1^) combined with aerobic respiration result in low concentrations of particulate (POC, about 0.4 wt% in the mixed layer and about 0.15 wt% below) and dissolved (DOC, about 200–400 μM close to the interface and about 100 μM below) organic matter (Fig. 2).

Authigenic enrichment of metals at the study sites were estimated using detrital correction with Ti assuming that the element/Ti ratio is the same as the upper continental crust^41^. Whether using Al or Ti for this correction makes little difference. For example, the estimated mean authigenic Nd concentration is 53 ppm using Al versus 51 ppm using Ti. There is little vertical change of elemental concentrations in the upper-sediment package because of bioturbation. The means of estimated authigenic Nd concentration in the upper 30 cm at Stations 3, 4 and 5 are 49 ± 5 ppm (1*σ*), 33 ± 7 ppm and 73 ± 9 ppm respectively, and the mean of all samples combined is 51 ± 18 ppm. This inter-site difference is entirely attributed to dilution by biogenic materials (mainly carbonate which is much higher at Station 4). The relative authigenic enrichment of Nd, expressed as the ratio of authigenic to total Nd, is nearly identical at all sites, and the mean ratio of all samples combined is 0.76 (±0.09, a relative standard deviation of about 10% which is comparable to analytical precisions). The result demonstrates a high degree of homogeneity of the authigenic sediments in the study region despite the strong surface-productivity gradient, consistent with biogenic particles contributing little to the particulate REE flux.

### REE carriers

Geochemical data of water-column particles and oxide-rich sediment phases were compiled and compared with our abyssal authigenic sediments. The paired particle Nd, Mn and Fe data shown in Extended Data Fig. 1a are available only from the GEOTRACES studies^25,38,60^. However, because few sites in the GEOTRACES dataset reported the full REE data, the particle REE data shown in Extended Data Fig. 1c mainly come from other literature sources^11,63,78–80^. In these studies, water-column particulate authigenic REE concentrations were either reported using chemical extraction^11,63^, or estimated here using detrital correction based on Al (ref. ^79^) or Th (refs. ^38,78,80^), depending on which measurement was available. The Mn–Fe crust and nodule data are from the National Oceanic and Atmospheric Administration and Minerals Management Service Marine minerals geochemical database^81^. The data of dispersed Mn–Fe oxides in marine sediments extracted by leaching sediments or foraminifera are compiled by ref. ^47^. The *K*_d_ of water-column particles were computed using the estimated authigenic REE concentration and paired seawater dissolved REE concentration from the same study. When computing *K*_d_ for crusts and nodules, we used the REE concentrations of our Pacific bottom water to represent deep-ocean seawater in general. When computing *K*_d_ for the authigenic sediment, we used the estimated authigenic REE concentrations and bottom-water dissolved REE concentrations from our study sites. Finally, we also compared our data with the Pacific sediment compilation of ref. ^82^ when showing the correlation between Nd and Mn concentrations in abyssal sediments (Extended Data Fig. 1d).

Previous studies have shown that phosphate is also an important host of REE in abyssal sediments^82,83^. This observation does not contradict our conclusion that oxides are the main carriers of REE in the water column and surface sediments. As pristine biogenic phosphates contain negligible amounts of REE^64^, the association of REE with phosphates can only happen post-deposition. Studies have demonstrated the diagenetic transfer of REE from oxides to phosphates on the Oregon margin using diagenetic modelling^15^, and on the abyssal seafloor using geochemical and mineralogical analysis^84^. Because this kinetic mass transfer is slow on the abyssal seafloor (>10^4^ years according to ref. ^84^), the association with phosphate manifests more strongly in the deeper sediment package, and in regions of lower sedimentation rate^83^. However, this mass transfer has little effect on the total solid sediment budget, and the total (oxide plus phosphate) authigenic REE burial flux remains similar to that of the oxide-carried particle rain rate^15^. For example, a much higher authigenic Nd concentration up to 300 ppm hosted in phosphate-rich abyssal sediments has been found in locations with a much lower sedimentation rate (<0.05 cm kyr^−1^)^83^, yet the implied burial rate is <40 pmol cm^−2^ yr^−1^, no more than at our sites and entirely supportable by the Mn oxide-hosted particle rain rate. Thus, we conclude that this diagenetic transfer reconciles the apparent dichotomy that scavenged REE reaching the abyssal seafloor is mainly hosted by Mn oxide, yet in deeply buried sediments phosphate can be the main carrier.

### Water-column and sediment mass fluxes

Given the uniformity of authigenic enrichment and detrital mineralogy, we assume that the fluxes of the non-biogenic (detrital and authigenic) materials are similar at the three sites. Our estimation of sediment burial flux uses data from Station 4 where radiocarbon dating is possible. The data here show a bioturbated depth of about 9 cm and a burial velocity of 0.53 cm kyr^−1^. The burial rate is computed as

3 $${F}_{{\rm{burial}}}=(1-\phi )\rho wM,$$

where *ϕ* is porosity (0.85 ± 0.04, 1*σ*, assuming 5% uncertainty), *ρ* is sediment grain density (2.65 ± 0.13 g cm^−3^, assuming 5% uncertainty), *w* is the burial velocity (0.53 ± 0.077 cm kyr^−1^), and *M* is the total (42 ± 1 ppm Nd) or authigenic (36 ± 1 ppm Nd) concentration in the mixed layer. Errors are propagated into the results.

To estimate the particle Nd rain rate, we combine the GEOTRACES particle concentration data^25^ with the Joint Global Ocean Flux Study (JGOFS) particle flux data^85–87^ based on sediment traps. The rain rate is computed as

4 $${F}_{{\rm{rain}}}={F}_{{\rm{p}}}{{\rm{Nd}}}_{{\rm{p}}}\,,$$

where *F*_p_ is the particle flux and Nd_p_ is the total or authigenic Nd concentration in particles. The JGOFS equatorial Pacific traps were deployed along approximately 140° W (15° S–15° N), slightly eastwards of our study sites (152° W, 3° N–11° N). The particle mass flux measured by the traps below 3,000 m at 5° N (between our Stations 4 and 5) is 64 ± 16 mg m^−2^ d^−1^. The GEOTRACES GP16 Station 36 (152° W, 10.5° S) is the closest site with particulate data available that has an oceanographic setting similar to our sites^25^. The total and authigenic Nd concentrations below 2,500 m (excluding the benthic nepheloid layer) in the sinking particles are 4.2 ± 1.3 ppm and 3.4 ± 1.2 ppm, respectively. The relative enrichment of authigenic Nd in the deep-ocean particles (about 80%) is similar to that in our abyssal sediments (about 76%).

### Diagenetic model

We performed diagenetic modelling using SedTrace^88^, a tool that automates the generation and simulation of trace-element diagenesis. The diagenetic equations of biogeochemical tracers and REE have been reported in our previous study of REE diagenesis on the Oregon margin^15^ as well as in the model description paper of SedTrace^88^. The model includes the following biogeochemical tracers: POC, DOC, CaCO_3_, O_2_, NO_3_, NH_4_^+^, TCO_2_ and H^+^. POC is modelled using a 2-G model, including a labial component (POC_1_) and a refractory component (POC_2_) suggested by previous works in the study region^77^. We assumed that the POC components are first converted to DOC (DOC_1_ and DOC_2_ respectively), which subsequently undergo aerobic remineralization^89^. We also included nitrification and carbonate dissolution in the model^15^. The pH model follows the standard construction in SedTrace^88^.

The biogeochemical model is tuned to fit the average sediment biogeochemistry represented by Station 5 (Fig. 2). To capture the high concentration of DOC at the sediment–water interface (Fig. 2d), the labial DOC_1_ (Extended Data Fig. 2b) needs to be a high-molecular-weight compound with low molecular diffusivity^90^ relative to the refractory DOC_2_, consistent with the conceptual model of POC remineralization in marine sediments^89^. Because the abyssal sediment is poorly buffered, modelled pH decreases from 7.7 at the sediment–water interface to 7.2 at 20 cm, qualitatively consistent with the measured values (about 7.2–7.3 at Station 5, although we note the limited utility of this data because of the sampling issue mention above; Extended Data Fig. 2c).

Three processes affect porewater REE in the model: complexation with dissolved ligands, release during POM remineralization and sorption onto oxides. Complexation of REE with inorganic ligands and the related stability constants follow that of refs. ^59,91^. We also include REE complexation with the two types of DOC, and the stability constants are tuned within the range of organic ligands reported in literature^92^ (Extended Data Fig. 3c). To model Nd by POM remineralization, we derive a reasonable estimate of the Nd/C ratio released by POM. We start from the water-column *K*_d_ of POM estimated above, and find a value that is consistent with this *K*_d_ and reasonably fits the porewater Nd profile. The release of the other REE is scaled to Nd, the REE pattern of which is tuned following the pattern of *K*_d_ of POM reported in the literature^93–95^ (Extended Data Fig. 3b). Finally, we implement pH-dependent sorption of REE onto Mn–Fe oxides following refs. ^59,96,97^, which was derived using laboratory adsorption experiments using synthetic oxides in ionic media similar to seawater. We assume that only the free REE ions are adsorbed, and use the pH-dependent free-ion-based $\genfrac{}{}{0ex}{}{{\rm{f}}}{}{K}_{{\rm{d}}}$, which is related to the total concentration-based *K*_d_:

5 $${K}_{{\rm{d}}}{=}^{{\rm{f}}}{\alpha }^{{\rm{f}}}{K}_{{\rm{d}}}\,,$$

where ^f^*α* is the fraction of free ion. *K*_d_ is an apparent constant that depends on the speciation of REE. We estimated $\genfrac{}{}{0ex}{}{{\rm{f}}}{}{K}_{{\rm{d}}}^{{\rm{N}}{\rm{d}}}$ using the $\genfrac{}{}{0ex}{}{}{}{K}_{{\rm{d}}}^{{\rm{N}}{\rm{d}}}$ derived from the GEOTRACES particle data and seawater speciation results, and found that it is about three orders of magnitude higher than the experimental values measured using synthetic Mn–Fe oxides in the lab, a known discrepancy^98^ that is likely attributed to the difference in the surface chemistry between natural and synthetic oxides and the uncertainty in the aqueous speciation of REE in seawater^59^. In an ad hoc approach, we used the lab-based REE pattern of $\genfrac{}{}{0ex}{}{{\rm{f}}}{}{K}_{{\rm{d}}}$ in the model, but scaling them such that the value of $\genfrac{}{}{0ex}{}{{\rm{f}}}{}{K}_{{\rm{d}}}^{{\rm{N}}{\rm{d}}}$ is consistent with the field data. That our model can successfully reproduce the porewater data REE pattern suggests that although the lab-based values of synthetic oxide $\genfrac{}{}{0ex}{}{{\rm{f}}}{}{K}_{{\rm{d}}}$ may not apply to natural oxides, their REE pattern appears like natural oxides. This implies that the discrepancy is caused by factors common to all REE, and thus most likely related to particle surface chemistry rather than REE speciation. To model porewater *ε*_Nd_ we added ^144^Nd and the radiogenic ^143^Nd as two individual tracers to the model^15^, both of which are subject to the same processes.

The decrease of pH reduces $\genfrac{}{}{0ex}{}{{\rm{f}}}{}{K}_{{\rm{d}}}$, whereas the increase of organic ligand concentration lowers ^f^*α*, thus decreasing *K*_d_ in sediment (Extended Data Fig. 2d), promoting relative desorption of REE from Mn–Fe oxides. Modelled REE speciation is shown using Nd and Lu to represent LREE and HREE, respectively (Extended Data Fig. 2e,f). *K*_d_ computed using equation (5) is shown in Extended Data Fig. 3a where the ribbon indicates the range of *K*_d_ in the sediment package. Modelled porewater REE patterns are shown by normalizing to either the local bottom water or Post-Archaean Australian Shale (PAAS) (Extended Data Fig. 3).

### Benthic fluxes

The benthic flux of REE is calculated as:

6 $${F}_{{\rm{benthic}}}=-\phi /(1-{\rm{ln}}{\phi }^{2})\sum _{{\rm{i}}}{D}_{{\rm{sw}}}^{i}{\partial C}^{i}\,/\partial z,$$

where *i* is the index of REE species, including the inorganic species and complexes with DOCs, ${D}_{{\rm{sw}}}^{i}$ is the molecular diffusivity and ∂*C*^*i*^/∂*z* is the concentration gradient. The benthic flux is computed by summing over the individual species because the organic and inorganic ligands have different molecular diffusivity^89^. This calculation is straightforward in the model as the individual species are modelled directly. For data-based estimates, we distinguish the low-diffusivity DOC_1_ from the other species that have the same diffusivity in the model. The porewater concentration profiles of REE can be considered as the superposition of a shallow peak (DOC_1_ complex) onto a slowly increasing background (DOC_2_ and inorganic species) concentration. We use the concentration immediately below the shallow peak to represent the background concentration, and the concentration of the DOC_1_ complex is calculated by subtracting the background from the measured profiles. ∂*C*^*i*^/∂*z* was then computed using the decomposed profiles. Given the small role of DOC_1_ complexation in the speciation of LREE, Nd is negligibly affected by this decomposing compared with HREE Lu. The data-based estimate was done using the mean concentration profile averaged over the three sites, and the errors are propagated throughout the calculation to give an overall estimate of benthic flux and its uncertainty in the study region.

Aside from measured and modelled benthic REE fluxes, in Extended Data Fig. 3 we also show the following sedimentary fluxes: data-based total sediment and authigenic REE burial fluxes, and POM-associated REE rain rates used in the model.

### Water-column model

We model the 3D distributions of Nd concentration using the transport matrix method^34,99^:

7 $${{\rm{\partial }}{\rm{N}}{\rm{d}}}_{{\rm{T}}}/{\rm{\partial }}t+{{\bf{T}}}_{{\rm{c}}}{{\rm{N}}{\rm{d}}}_{{\rm{T}}}+{{\bf{T}}}_{{\rm{w}}}{{\rm{N}}{\rm{d}}}_{{\rm{s}}}={F}_{{\rm{r}}{\rm{i}}{\rm{v}}{\rm{e}}{\rm{r}}}+{F}_{{\rm{d}}{\rm{u}}{\rm{s}}{\rm{t}}}+{F}_{{\rm{b}}{\rm{e}}{\rm{n}}{\rm{t}}{\rm{h}}{\rm{i}}{\rm{c}}}\,,$$

where Nd_T_ is the total Nd concentration, and the dissolved (Nd_d_) and scavenged Nd (Nd_s_) are computed using equation (1) assuming reversible scavenging; **T**_c_ and **T**_w_ are the transport matrices for 3D ocean circulation and vertical particle sinking respectively; and *F*_river_, *F*_dust_ and *F*_benthic_ are the per volume sources of Nd due to river input, dust deposition and benthic flux, respectively. Other sources are considered minor and not included^9,13,34,100^. We create and solve the steady-state model using the Julia language package AIBECS.jl^99^, which is similar to the popular AWESOME OCIM framework of ocean biogeochemical modelling^101^. Our model is inspired by previous models of marine Nd cycles^3,13,100,102^, especially GNOM^34^, but diverge from them in the implementation of the particle scavenging and benthic fluxes, as described in this study. We give a brief description of our model construction and experiments here.

The Ocean Circulation Inverse Model (OCIM)^43,103^ has been widely used as the circulation matrix **T**_c_ in OBMs of trace elements^34,101^. Here we use the OCIM2-48L version (2° horizontal resolution, 48 vertical layers), which updates the previous versions by adding more depth layers and implementing bottom-intensified mixing following the state-of-the-art parameterization of ref. ^16^. The result is a better representation of the abyssal Pacific circulation strongly shaped by seafloor topography^43^, making this model version ideal for studying the bottom-up control on the marine Nd cycle.

Dissolved, scavenged and total Nd concentrations are related via *K*_d_ (equation (1)). In our model, we only include biogenic particles and MnO_2_. We obtained a 3D field of POM from previous OBMs^101^ and mapped it onto the model grid. We obtained 3D fields of carbonate and opal concentrations using satellite-derived surface export flux and depth-dependent attenuation laws following previous models (remineralization length scales were set to 3,500 m for carbonate and 10,000 m for opal)^13,104^.

We currently lack a faithful reconstruction of the 3D field of MnO_2_ by OBMs owing to the challenge of modelling the marine Mn cycle^54^. Instead, we use an artificial neural network (ANN) to interpolate the GEOTRACES particle data (IDP 2021 Version 2)^38^ onto our model grid, as previous studies have done for other ocean biogeochemical fields^31,105^. The data are mainly from the Pacific (GP16) and Atlantic (GA01, 03, 06), with minor contributions from Arctic (GN01, 02, 03) and Southern Ocean (GA10) cruises. This ANN is a perceptron consisting of 2 hidden layers with 50 neurons and rectified linear unit activation function. We train with the following predictors: sampled water depth and latitude; temperature, salinity, oxygen, phosphate and silicate concentrations from World Ocean Atlas^56^; mixed-layer depth, Δ^14^C, POM concentration and ^3^He/^4^He ratio from previous OCIM studies^43,101,103^; and transmissometer-derived nepheloid-layer particle concentration^101,106^. These predictors are selected to account for potential locational, physical and biogeochemical factors controlling the distribution of MnO_2_, as well as the influence of a hydrothermal source and sediment resuspension. The gridded data products of these predictors are interpolated at the GEOTRACES sample locations and model grid points. We randomly selected 80% of the data from each ocean basin to create a training dataset, and the residual 20% was used for testing. In this way, the ANN results are less biased towards any individual ocean basin. Such data partitioning was repeated to generate a 500-member ensemble. We trained the ANN with the Adam optimizer and L_2_ regularization using the Julia machine-learning package MLJ.jl^107^. The model misfit generally stops changing after about 50 epochs, and we terminate the training after 100 epochs. The correlation coefficients between the predicted and observed MnO_2_ are 0.92 and 0.90 (in the log-transformed space) for the training and testing sets, respectively (Extended Data Fig. 6). The root mean square errors are 0.25 µg l^−1^ for both sets. The generated MnO_2_ field correctly captures the main features in the GEOTRACES observation^38^ including, for example, the hydrothermal plume, nepheloid layers and oxygen minimum zones in the Pacific GEOTRACES transect GP16^25^, and the strong benthic nepheloid layer in the Northwest Atlantic^60,106^ (Extended Data Fig. 6).

The river discharge in the model is derived from a global discharge model^108^, and the river dissolved Nd concentration is from the observational dataset of ref. ^109^. We assume a 30% removal of river Nd flux in the estuary^9,110^. Dust depositional flux in the model is derived from a global dust model^111^. We assigned a dust Nd concentration of 27 ppm (similar to the upper continental crust) and solubility of 2%, as in previous models^9,13^.

We treated the benthic flux per unit area as a function of water depth (Fig. 3e) and tuned it within the range of the observations in the model experiments. The published benthic flux data are derived using porewater profiles^44,68^ and generally consider only the diffusive fluxes, which may underestimate the total benthic flux by ignoring other transport mechanisms such as bio-irrigation and advection^15,68^. Ocean models, even the improved OCIM2-48L, generally cannot entirely capture ocean bathymetry owing to limited resolution. We use the ETOPO 2022 global relief model to derive a subgrid bathymetry that better captures the distribution of the area-to-volume ratio in the real ocean^99^, and allows the conversion of benthic flux per unit area to benthic flux per unit volume (Fig. 3e,f and Extended Data Fig. 7).

Through sensitivity tests (Fig. 3 and Extended Data Figs. 4 and 5), we evaluated the potential impact of particle scavenging and benthic flux on the distribution of Nd concentration in the Pacific. In the first experiment, we do not consider benthic flux and only include POM as a scavenger. We use a *K*_d_ of 10^5.8^ for POM, within the range of our data-derived values, and a settling velocity of 2.0 m per day similar to Th-based estimates^2,112–115^. Although our analysis of the GEOTRACE particle data do not support opal and carbonate as important Nd scavengers, we still chose to perform model tests to be comparable with previous reversible scavenging models. In these tests, we set the *K*_d_ to 10^7.0^ and 10^5.6^ for opal and carbonate, respectively, and these values were chosen to fit the surface Nd concentrations (Extended Data Fig. 4). In the second experiment, we further added MnO_2_ scavenging with a *K*_d_ of 10^8.7^ (Extended Data Fig. 6), according to our data-derived value, while keeping other parameters the same. In the last experiment, we further added a benthic flux while keeping other parameters the same. We used a vertical profile of benthic Nd flux per area that is consistent with observations, which is converted to flux per volume using the seafloor-area-to-volume ratio in the model subgrid bathymetry (Fig. 3e,f and Extended Data Fig. 7).

The model equation for radiogenic ^143^Nd is similar to that of total Nd (equation (9)), except that the sources on the right-hand side are converted to sources of ^143^Nd according to their isotope compositions. We obtain the *ε*_Nd_ of river input according to the gridded continental rock *ε*_Nd_ dataset of ref. ^13^. We map the *ε*_Nd_ of dust based on dust provenance^13,111^.

Previous studies often used the gridded global sediment detrital *ε*_Nd_ map^9^ to represent benthic flux *ε*_Nd_. However, it is well recognized that detrital sediment *ε*_Nd_ do not always represent the *ε*_Nd_ of benthic flux^9,13,33,47^, which is sometimes dominated by a small fraction of reactive detritus, such as volcanic materials in the Pacific^14,15,33^. Consequently, such models have difficulty in explaining Pacific seawater *ε*_Nd_ data^13^. Other studies have attempted to modify the detrital *ε*_Nd_ map to solve this problem^34^, but such modifications are generally arbitrary, and not constrained by studies of sedimentary Nd cycling.

Here we parameterize the benthic flux *ε*_Nd_ following our present and previous^8,15,33,47,116^ observational and modelling studies of sediment diagenesis. In the Pacific, we partition the benthic flux used in the Nd concentration model above into a recycled component and a new component. The *ε*_Nd_ of the recycled flux is set to be the same as seawater *ε*_Nd_ at the bottom-most water-column grid above the seafloor. The *ε*_Nd_ of the new flux is set to be pure volcanic (+10 *ε*). We use this value for sedimentary volcanics as a starting point for our sensitivity test. Our model choice of the proportion of the new source (0–30%) was based on porewater and authigenic sediment data^15,116^ (Fig. 4b). If we set the new source *ε*_Nd_ to lower (higher) values, the porewater and authigenic sediment data would simply require a greater (lower) proportion of the new source, but the net effect of the new flux would remain the same. There is no observational study of benthic flux *ε*_Nd_ in other ocean basins, so outside of the Pacific we set the benthic flux *ε*_Nd_ to be the same as detrital sediment *ε*_Nd_ compiled by ref. ^9^ (Extended Data Fig. 8). Previous research has shown that using detrital *ε*_Nd_ to represent benthic flux *ε*_Nd_ works reasonably well in the other basins^13^. Our model results that the Southern Ocean *ε*_Nd_ can be reproduced (Fig. 4) shows that this approach is adequate given our focus on the Pacific Ocean, and we leave the study of benthic fluxes for the future.

In a set of sensitivity tests, we set the new component to be 0%, 10%, 20% and 30% of total benthic flux, respectively (Extended Data Fig. 9). We use the modelled total Nd concentration from the third experiment in the concentration sensitivity test (Fig. 3b). We model the concentration of radiogenic ^143^Nd, and compute the *ε*_Nd_ letting ^143^Nd and ^144^Nd sum to the modelled total Nd.

We also tested model sensitivity to where the new benthic flux is applied. In the test shown in Fig. 4, the new flux is added to all seafloor areas in the Pacific. In Extended Data Fig. 10a–c, we limit the new benthic flux to the margins up to water depths of 1,000 m, 2,000 m and 3,000 m, respectively. In Extended Data Fig. 10d–f, we restrict the new flux to the abyssal seafloor below 3,000 m, either in the entire Pacific, or only in the South Pacific, or everywhere except the dust province in the North Pacific where detrital sediment is predominantly composed of Asian dust^48^. The areal extent of the North Pacific dust province is delimited by the contour of detrital sediment *ε*_Nd_ of −8 (Extended Data Fig. 8).

Finally, we model the water-mass age (that is, ideal age) following the original OCIM2_48L study^43^. The modelled circulation age is used to show the impact of benthic exposure time^33^ on the distribution of seawater *ε*_Nd_.

### Bottom-intensified mixing

The diapycnal diffusivity *κ*_*ρ*_ in Extended Data Fig. 7c is computed using the state-of-the-art parameterization of topographic mixing driven by internal tide dissipation from ref. ^16^, which has been validated against microstructure observations. The horizontal mean depth profile is:

8 $${{\kappa }}_{{\rho }}=\langle B\rangle /\langle {N}^{2}\rangle =\langle {\varepsilon }/6\rangle /\langle {N}^{2}\rangle ,$$

where *B* is buoyancy flux, *N* is buoyancy frequency, *ε* is turbulence production and ⟨⟩ denotes these are horizontally averaged. The diapycnal diffusion length of Nd is then:

9 $${L}_{{\rm{Nd}}}=2{({\kappa }_{\rho }{\tau }_{{\rm{Nd}}})}^{1/2},$$

The diffusion length *L*_Nd_ (Extended Data Fig. 7d) should be interpreted as the length scale that a source of Nd can be of influence by turbulent diffusion alone in the water column on the timescale of Nd residence time (*τ*_Nd_). Literature reports of *τ*_Nd_ are in the range of about 350–750 years^3,9,34,44,102,117^, and we use a value of 400 years as a first-order estimate.

### Nd data compilation

We compiled literature data of seawater, core-top authigenic and detrital sediment *ε*_Nd_, and seawater Nd concentration previously^9^, which is updated here to include recent GEOTRACES results^23,38,118^ (Extended Data Fig. 8). The paired authigenic and seawater data shown in Fig. 4b were created by binning the Pacific data into boxes that have 10° latitudinal, 20° longitudinal and 300 m (about 0–1,500 m) or 1,000 m (below 2,000 m) vertical resolutions. Data that fall into the same box were averaged and the standard deviations are reported (Fig. 4b).

We also compiled Nd concentration data in organic-rich sediments from the literature^55,116,119–121^ to complement other trace-metal data from ref. ^49^, shown in Fig. 5d.

## Online content

Any methods, additional references, Nature Portfolio reporting summaries, source data, extended data, supplementary information, acknowledgements, peer review information; details of author contributions and competing interests; and statements of data and code availability are available at 10.1038/s41586-025-09038-3.

## Data Availability

The geochemical data generated by this study are available at the Biological and Chemical Oceanography Data Management Office (10.26008/1912/bco-dmo.928400.1, 10.26008/1912/bco-dmo.928152.1, 10.26008/1912/bco-dmo.928246.1). GEOTRACES data are available at British Oceanographic Data Centre (10.5285/cf2d9ba9-d51d-3b7c-e053-8486abc0f5fd). ETOPO 2022 bathymetry is available from NOAA National Centers for Environmental Information (10.25921/FD45-GT74). Global tidal mixing data are available at 10.17882/73082. The NOAA and MMS Marine Minerals Geochemical Database is available at 10.7289/V52Z13FT. GLODAP (v2.2022) Pacific seawater δ^13^C data are available at 10.25921/1f4w-0t92. The global Nd data compilation is available at 10.5281/zenodo.10859057 (ref. ^122^).

## Code availability

The source code of SedTrace is available on Zenodo at 10.5281/zenodo.7225861 (ref. ^123^). The diagenetic model of REE is available on Zenodo at 10.5281/zenodo.10847073 (ref. ^124^). The water-column Nd cycle model is available on Zenodo at 10.5281/zenodo.10847786 (ref. ^125^).
